# Multiple Origins of Extracellular DNA Traps

**DOI:** 10.3389/fimmu.2021.621311

**Published:** 2021-02-24

**Authors:** Edgar Ramos-Martínez, Leticia Hernández-González, Iván Ramos-Martínez, Laura Pérez-Campos Mayoral, Georgina I. López-Cortés, Eduardo Pérez-Campos, Gabriel Mayoral Andrade, María Teresa Hernández-Huerta, Marco V. José

**Affiliations:** ^1^ School of Sciences, Benito Juárez Autonomous University of Oaxaca, Oaxaca, Mexico; ^2^ Biochemistry and Immunology Unit, National Technological of Mexico/ITOaxaca, Oaxaca, Mexico; ^3^ Glycobiology, Cell Growth and Tissue Repair Research Unit (Gly-CRRET), Université Paris Est Créteil (UPEC), Créteil, France; ^4^ Research Centre Medicine UNAM-UABJO, Faculty of Medicine, Benito Juárez Autonomous University of Oaxaca, Oaxaca, Mexico; ^5^ Theoretical Biology Group, National Autonomous University of Mexico, Mexico City, Mexico; ^6^ CONACyT—Faculty of Medicine, Benito Juárez Autonomous University of Oaxaca, Oaxaca, Mexico

**Keywords:** extracellular DNA traps, evolution, multicellular organisms, extracellular neutrophil traps, neutrophils

## Abstract

Extracellular DNA traps (ETs) are evolutionarily conserved antimicrobial mechanisms present in protozoa, plants, and animals. In this review, we compare their similarities in species of different taxa, and put forward the hypothesis that ETs have multiple origins. Our results are consistent with a process of evolutionary convergence in multicellular organisms through the application of a congruency test. Furthermore, we discuss why multicellularity is related to the presence of a mechanism initiating the formation of ETs.

## Introduction

Two of the evolutionarily conserved defense mechanisms in multicellular organisms are coagulation (1, 2) and the formation of extracellular traps. We have reviewed the latter.

Brinkmann et al. (3) observed that, during inflammation and after stimulation with phorbol myristate acetate (PMA), lipopolysaccharide (LPS) and interleukin 8 (IL-8), neutrophils form decongestant chromatin structures studded with microbicidal proteins, mainly elastase and histones. These structures are networks that trap and kill bacteria and are named Extracellular Neutrophil Traps (NETs). In addition to neutrophils, certain cells form extracellular DNA traps (ETs), for example, monocytes, mast cells, and also eosinophils in mammals, heterophils in birds and hemocytes in arthropods, mollusks, and crabs (4, 5). In plants, root border cells form extracellular root traps (RETs) (6), and in the protozoan, *Dictyostelium discoideum*, ETs have been described in their multicellular aggregative phase (7).

Firstly, we review the findings on ETs in different organisms to compare their similarities. Following this, we posit that ETs are a defense strategy that emerged early in the evolutionary history of eukaryotes. We examine the current evidence to discern whether ETs have independent origins in different taxa, or whether they are present in distant but related taxa and from a common origin. Finally, we discuss the supporting evidence as to why ETs are a multicellular defense strategy.

## Organisms That Produce Extracellular Traps

### Extracellular Traps in Protozoans

The amoeba *D. discoideum* (slime mold) is a protozoan that, in its natural habitat, is free-living, however, under laboratory conditions, food shortage induces the formation of aggregates of approximately 10,000 cells. The aggregate of *D. discoideum* is a migratory structure or “slug” consisting of specialized groups of cells. This stage of its development can be maintained under controlled conditions for 48 h, until it experiences terminal differentiation culminating in a fruiting body and a mass of spores supported by a stem (8).

One group of specialized slug cells, are sentinels cells (S cells), which perform immunological-like functions such as engulfing bacteria and sequestering toxins (9). These cells were shown to produce ETs following stimulation with bacteria and LPS in a reactive oxygen species (ROS) dependent manner by the activation of Nicotinamide Adenine Dinucleotide Phosphate-Oxidases (NADPH oxidases) A, B and C (homologous to human Nox2) (7). This signaling mechanism involves the TirA protein, which contains a Toll/Interleukin-1 receptor domain. The formation of ETs by S cells does not compromise cell viability since they are formed from mitochondrial DNA (mtDNA). The ETs trap particles and bacteria and can cause the death of the bacterium *Klebsiella pneumoniae* (7).

### Extracellular Traps in Plants

The defense of plants is comprised of various biochemical interactions, many of which occur in the extracellular environment as the apical zone of the roots is resistant to multiple pathogens (10). This resistance mechanism seems to involve a mucilaginous matrix of polysaccharides in separating “border cells” (11, 12). *Arabidopsis thaliana* border cells recognize molecular patterns of pathogens (PAMPs) such as peptidoglycans and flagellin, and increase the production of ROS (13, 14). The production of ROS is a common pathway of defense against pathogens in many organisms (15)

Among the defense mechanisms used by the border cells to combat possible pathogens, is the release of RETs (11). RETs consist of an extracellular DNA matrix, which was demonstrated by treating pea root tips (*Pisum sativum* ‘Little Marvel’) with endonuclease DNase I. This increased the susceptibility of the roots to infection by the fungus *Nectria haematococca* (6). Although RETs are continuously released when border cells disperse into the rhizosphere from root calyptra (16), their release can be amplified in response to microbial infections. For example, the border cells of *P. sativum* and tomato (*Solanum lycopersicum*) release RETs in response to the plant’s pathogenic bacteria, *Ralstonia solanacearum* and the fungus *N. haematococca*. In contrast, non-pathogenic bacteria such as *Escherichia coli*, *Sinorhizobium meliloti*, and *Pseudomonas aureofaciens* do not lead to the formation of RETs (17). The RETs of *P. sativum* and *S. lycopersicum* consist of extracellular DNA (exDNA), derived from DNA strands, polysaccharides and microbicidal proteins such as histone H4 (90% similar in base sequence to H4 histones of mammals) (17).

Like NETs, exDNA is a fundamental component of the structure of RETs and renders stability to the proteins that are released by the border cells (16). Together with the exDNA, numerous molecules are released in the RETs, for example, the glycosylated protein arabinogalactane (18), defensins (19), extensins (20), and xyloglucan (21). One study showed that more than 100 proteins are released by the cells of the root boundary (22). However, it has not been established which of these proteins are part of the RETs and which are released as part of other secretion processes.

The presence of ROS has been suggested in RETs. In border cells of *A. thaliana* and in *Linum usitatissimum* (flax), stimuli with flagellin (flg22) and peptidoglycan increase the production of ROS, for example hydrogen peroxide (H_2_O_2_) and singlet oxygen $\left(O_{2}^{-}\right)$. In addition, expression of the RbohD gene encoding a NADPH oxidase increases (23). Among the extracellular root proteins secreted by *A. thaliana* and *Brassica napus*, enzymes that produce ROS, such as copper-zinc superoxide dismutases and class III peroxidase, have been detected (24). In *Zea mays* the presence of superoxide dismutases has also been reported in the mucilage secreted by calyptra cells into the rhizosphere (25). However, further studies are required to assess whether these enzymes are integrated into ETs or are secreted by border cells in processes unrelated to RET formation. In addition, an evaluation of the activation of NADPH oxidases and the production of free radicals is required to assess it as a mechanism by which the release of RETs is stimulated, similar to NETs.

RETs perform similar functions to NETs, such as immobilizing and eliminating bacteria and fungi and limiting the dispersion of microorganisms within root tissues. They also maintain an optimal concentration of the proteins secreted by preventing their diffusion into the environment surrounding the root (12, 17). The presence of these RETs has been reported in a wide variety of plants (**Table 1**). However, there are still several issues that need to be evaluated. For example, it has been observed that border cells are continuously produced in the root cap or calyptra and are released toward the rhizosphere, but they have not been shown to move toward sites of infection (21). Border cells have been described as remaining viable when releasing RETs (16), but it has not been established whether the DNA is nuclear or mitochondrial, nor the mechanism by which it is released. RETs and NETs share many structural and functional characteristics in common. Driouich et al. (21) reviewed these similarities recently, which we can confirm with the following sentence from the article: “It is striking how remarkably similar RETs and NETs are in terms of composition and functional convergence”.

**Table 1:** Extracellular traps in reported species.

Phylum	Class	Reported species	Cells who produce them	ROS production	Components	Reference
Amoebozoa	Dictyostelia	*D. discoideum*	S cells	NADPH oxidase-dependent	mtDNA	(7)
Tracheophyta	Magnoliopsida	*P. sativum* *Z. mays* *A. thaliana* *S. lycopersicum*	Root border cells	Not determined	exDNA, H4 histone, Cytoplasmic proteins including antimicrobial proteins and polysaccharides	(17, 21); (26)
Mollusca	Bivalvia	*Crassostrea gigas*	Hemocytes	NADPH oxidase-dependent	DNA, H1-like and H5-like histones	(27)
Annelida	Clitellata	*Eisenia andrei*	Coelomocyte	NADPH oxidase-dependent and NADPH oxidase-independent	DNA, H3 histone	(28)
Arthropoda	Malacostraca	*Litopenaeus vannamei*	Hemocytes	NADPH oxidase-dependent	DNA, histones, C-type lysozyme	(29)
		*Marsupenaeus japonicus* *Carcinus maenas*				(30, 31)
Chordata	Actinopterygii	*Scophthalmus maximus* *Danio rerio* *Pimephales promelas* *Cyprinus carpio*	Neutrophil	NADPH oxidase-dependent	DNA, H2A and H2B histones, elastase, MPO	(32) (33–35)
Chordata	Aves	*Gallus gallus domesticus*	Heterophils	NADPH oxidase-dependent	DNA, histone, elastase	(36)
Chordata	Mammalia	*Mus* *musculus* *Bos taurus* *Capra sp* *Phoca vitulina* *Canis* sp. *Felis catus* *Hommo sapiens*	Neutrophil	NADPH oxidase-dependent and NADPH oxidase-independent	mtDNA, and nuclear DNA, H1, H2A, H2B, H3 and H4 histone, MPO, neutrophil elastase (NE), lactoferrin, tryptase	(3, 37–39) (40, 41)
			Mast cell	NADPH oxidase-dependent	DNA, tryptase, histones	(42)
			Eosinophil	NADPH oxidase-dependent	mtDNA, nuclear DNA mitochondial proteins, MBP	(43)
			Basophils	NADPH oxidase-independent	Granule proteins, mtDNA	(44)
			Lymphocytes	Not determined	mtDNA	(45)
			Monocyte/macrophage	NADPH oxidase-dependent and NADPH oxidase-independent	mtDNA, nuclear DNA, histones, MPO, antimicrobial peptides	(46)

### Extracellular Traps in Invertebrates

Invertebrate animal cells can form ETs. Hemocytes are the cells responsible for the immune response in arthropods. Shrimp hemocytes, (*Marsupenaeus japonicus* and *Litopenaeus vannamei*) form ETs with DNA and type C lysozymes in response to PMA, LPS, peptidoglycan and *E. coli* (29, 30). The crustacean, *Carcinus maenas*, forms ETs in response to LPS and *Listonella anguillarum*, a pathogenic bacterium in crustaceans (31). Hemocytes of the mollusk, *Crassostrea gigas*, form ETs with DNA fibers and histone-like proteins, H5-like and H1-like, in response to tissue damage and infection by the pathogenic bacterium *Vibrio tasmaniensis* LGP32. This mechanism depends on the accumulation of ROS by the action of NADPH oxidase. In some experiments, the hemocytes of *C. gigas* do not form ETs in response to PMA, as occurs in vertebrates (27).

Coelomocytes are the immune defense cells in annelids. *Eisenia andrei* coelomocytes form ETs both *in vivo* and *in vitro* in response to PMA, Gram positive bacteria, Gram negative bacteria, LPS and fungal polysaccharides. *E. andrei* ETs contain DNA and histone H3, the formation of coelomocyte ETs in response to PMA is ROS-dependent, although the formation of ETs in response to *Xenorhabdus bovienii* is independent of ROS (28).

### Extracellular Traps in Fish and Birds

Zebra fish (*Danio rerio*) neutrophils form ETs with DNA, myeloperoxidase (MPO) and elastase in response to PMA, a calcium ionophore and β-glucan, like mammalian neutrophils (33). *Scophthalmus maximus* neutrophils form ETs containing DNA and histones in response to LPS, and *in vivo* infection, these ETs trap *E. coli* and *Pseudomonas fluorescens.* ETs kill *E. coli* but not *P. fluorescens* (32). Carp neutrophils (*Cyprinus carpio*) form ETs containing DNA and histones H2A, H2B (47). The equivalent cells in birds are heterophiles. In birds, these cells form ETs that contain DNA, histones and elastase in response to hydrogen peroxide and PMA (36, 48).

### Extracellular Traps in Mammals

ETs were first described in human cells, specifically from neutrophils (3). Since Brinkmann’s study, several publications have revealed that this defense mechanism is not exclusive to neutrophils, as other immune cells are capable of ejecting either nuclear or mitochondrial DNA from the extracellular space. Traps, described as sticky fibers, are made up of DNA, granular proteins and enzymes, and it is likely that ETs could function as immune regulators for the inflammatory response (49).

#### Neutrophil Extracellular Traps

Different antimicrobial processes are known in neutrophils, e.g. phagocytosis, the killing of invading pathogens, and degranulation (50, 51). Phagocytosis is a receptor-mediated process that involves the internalization of particles and its subsequent fusion with lysosomes to form a phagosome (52). Another important anti-microbial mechanism is the release of ETs. This was identified in 2004 (3). NETs are involved in the immune defense against pathogens, inflammation (53), thrombosis (54), autoimmunity (55), and cancer (56).

These NETs are fibers composed of nuclear or mitochondrial DNA, microbicidal molecules such as histones (H1, H2A, H2B, H3, and H4) (57), the antimicrobial peptide LL-37 (58), neutrophil elastase, cathepsin G, proteinase 3, lactoferrin, tryptase, gelatinase, MPO and cytoplasmic proteins such as tubulin (59).

Several mechanisms have been identified in the formation of NETs, which depends on experimental conditions. *In vivo* NET formation (60), known as NETosis, involves neutrophil death and the accumulation of ROS produced by NADPH oxidase. In addition, there is disruption in internal membranes and cytosolic mixing. Extrusion of NETs occurs in a manner dependent upon the MPO and the necroptotic cell death effector “mixed lineage kinase domain-like” (MLKL). The formation time of NETs in this pathway is lengthy (2–3 h) (61).

In the vital formation of NETs, the neutrophil continues to retain chemotactic and antimicrobial functions after extrusion of the ETs. Several types of vital NET formation have been described (62). In 2009, Yousefi et al. (63), showed that, at low LPS concentrations, neutrophils appear to release NETs with mtDNA, and these neutrophils survive longer than unstimulated neutrophils. NETs are reported to form in response to saliva in L-selectin dependent manner, independent of NADPH oxidase and elastase. These NETs are more resistant to the action of DNases (64). In the formation of NETs by the independent way of NADPH oxidase, ROS come from the mitochondria and the blockade of MPO does not inhibit the extrusion of NETs, although it is partially dependent on MLKL (61, 65). Processes such as vesiculation, DNA decondensation, release of nuclear DNA into the cytoplasm and expulsion of extracellular DNA can be observed in this pathway (66).

Platelets activated by bacteria induce the extrusion of NETs in an integrin-dependent pathway with αIIbβ3, P and L selectins. This mechanism leading to the formation of platelet thrombosis. The interaction of NETs and proteins from the coagulation cascade generates a state known as thromboinflammation (67, 68).

Ultraviolet radiation induces the formation of NETs independently of NADPH oxidase, but at the same time, causes apoptosis. Therefore, this process is known as “ApoNETosis”. Histone citrulination does not occur in this mechanism (69). In some NET formation pathways, chromatin decondensation is related to increased transcription (70) and histone citrulination by peptidyl arginine deiminase 4 (PADI4). PADI4 is an enzyme that converts arginine to citrulline in histones, reducing their positive electrical charge and relaxing their bond to DNA to promote chromatin decondensation. PADI4 has been shown to intervene in the extrusion of NETs in both ionomycin, PMA and *Candida albicans* stimulated neutrophils in mice and humans. However, its involvement in NET formation is poorly understood (71).

Neutrophils of humans, rodents (72), bovines, ovines (38), pinnipeds, canines (4, 73), and felines (41), can form NETs in response to microorganisms, such as *Leishmania amazonensis*, *C. albicans*, *Toxoplasma gondii*, and *Plasmodium falciparum*, or in response to viruses such as respiratory syncytial virus, human immunodeficiency virus 1 and influenza virus (74–77). In addition, LPS, peptidoglycan, flagellin, the protein kinase C activator, PMA, and gold and silver nanoparticles can induce the formation of NETs (38, 78).

NETs’ principal function relies on its defense from pathogens, which is strikingly effective due to the high number of antimicrobial proteins. Moreover, pathogens enhance inflammation, affecting migration and coagulation, and vessels’ properties. In some cases, the formation of NETs can contribute to organic damage and induce thrombosis, for example, in SARS-CoV-2 infection, patients present elevated levels of cell-free DNA, DNA-associated MPO, citrullinated histone H3 and NETs (79, 80). It has been suggested that these NETs contribute to immunothrombosis in the acute respiratory distress syndrome caused by SARS-CoV-2 (81, 82). Furthermore, they hypothesize that the wrong clearance of extracellular DNA could lead to autoimmune diseases (83, 84).

#### Eosinophil Extracellular Traps

Eosinophils are the cells of the immune system specializing in defense against parasitic helminths. They are also associated with allergic and autoimmune diseases (85). Eosinophil ETs consist of nuclear or mtDNA and major basic protein (MBP) and eosinophil cationic protein (ECP). Unlike NETs, intact granules can be seen on Eosinophil ETs (86). The formation of ETs in eosinophils depends on ROS and NADPH oxidase and could end in cell death. ET release in eosinophils involves the dissolution of its bilobed nucleus, the rupture of the nuclear membrane, the mixing of cytosolic components and the rupture of the cell membrane (87). Yousefi et al. (88) showed that eosinophils form mitochondrial ETs a few seconds after stimulation with LPS, eotaxin, complementary factor C5a and *Escherichia coli.* In eosinophils primed with IL-5 or IFN-γ, the presence of the ATP-synthase gene subunit 6 (Atp6) in the released DNA was observed, although no nuclear proteins (43, 89).

#### Mast Cell Extracellular Traps

Mast cells are multifunctional cells of the mammalian immune system. Most of the mast cells are found in the skin and mucosa of the respiratory and gastrointestinal tracts, they modulate the function of other cells and, in infections, they can exert direct microbicidal functions (90). In 2008, mast cells were reported to can form ETs (91). Mast cells can form ETs in response to *S. pyogenes*, *C. albicans*, *E. faecalis*, *L. monocytogenes*, *P. aeruginosa*, and *Leishmania sp.* (42, 92, 93). The ETs of these cells contribute, along with other ETs, to atherosclerosis plaques developing the disease rapidly (94). Mast cell ETs are composed of DNA, histones, and tryptase, which is a specific protein in mast cells (91). The cathelicidin-derived antimicrobial peptide LL-37 (CRAMP/LL-37), mediators TNF-alpha, IL-17 and CXCL2 chemokine are all present in mast cell ETs, which are key in inflammation (95). Mast cells, like other cells, also need NADPH oxidase activation and the participation of the transcriptional factor hypoxia- inducible factor 1α (HIF- 1α) (43).

#### Basophil Extracellular Traps

Basophils are cells associated with inflammation, immunoregulation, allergic response and protection against parasites. Basophils release mtDNA extracellular traps by an independent NADPH oxidase mechanism (44). Basophil ETs contain mitochondrial, but not nuclear DNA. The mechanism occurs in the absence of functional NADPH oxidase; possibly the ROS necessary for the formation of basophil ETs come from the mitochondria. After activation of basophils by activation of the immunoglobulin E receptor or C5a complement receptor, the mitochondria and granules are concentrated at the extrusion site. While the nucleus remains in place, the histones are not part of the basophil ETs (96).

#### Monocyte Extracellular Traps

Monocytes are antigen-presenting immune cells (APCs) which contribute to immune defense with different mechanisms, such as phagocytosis, cytokine secretion, antigen presentation and tissue repair (97). Monocytes form ETs consisting of DNA, elastase, lactoferrin, MPO and citrullinated histones (46, 98). Peptidyl arginine deiminase 2 (PAD2) catalyzes the citrullination of histones in both subpopulations of human monocytes, the classical CD14+ CD16− and the non-classical CD14+ CD16+ (99). Citrullinated histones were observed in the nucleus and in the released DNA strands (98).

PMA, calcium ionophore A23187, platelet activating factor and zymosan have all been reported to trigger the release of ETs in monocytes (98). In addition, in *in vitro* studies, it has been observed that human sperm in the presence or absence of uropathogenic *E. coli* stimulate the release of ETs into peripheral blood monocytes (100). Similarly, *Staphylococcus aureus* cell-free culture supernatant also stimulates the release of ETs (101). Peripheral blood monocytes expel ETs within 10 min of exposure to supernatants containing NET components. This stimulus appears to be mediated by proteins such as elastase and citrullinated histones, and not by the DNA of neutrophil ETs (101). In this study, monocytes had no nucleus after the formation of ETs, and released cytoplasmic and nuclear components. The release of ETs in the monocytes was carried out both by the vesicular and the classical routes. The formation of monocyte ETs in response to NETs has been related to their neutrophil-cleansing function in apoptosis and NETs (101).

ET release in monocytes depends on the respiratory burst, since the NADPH oxidase 2 inhibitor, diphenylene iodonium chloride (DPI), inhibits ET release in PMA-stimulated monocytes. However, treatment with DPI has no effect on the release of ETs in A23187-treated monocytes, suggesting an alternative induction mechanism. Furthermore, inhibition of MPO and actin filament polymerization does not affect the release of ETs into monocytes (98). In studies conducted on caprine monocytes, it was observed that *Neospora caninum* stimulates the release of ETs. This process involves the activation of extracellular-signal-regulated kinase 1 and 2 (ERK 1-2) and p38, in addition to the production of ROS, since the inhibition of NADPH oxidase or MPO significantly reduced the formation of monocyte ETs (102).

#### Macrophage Extracellular Traps

Macrophages are cells specializing in the maintenance of hemostasis, regulation and tissue repair and immune response. They may originate from monocytes or from precursors residing in various tissues (103). Macrophage cell lines, monocyte derived macrophages, and alveolar bovine macrophages form macrophage ETs. Monocyte-derived macrophages release ETs when exposed to MPO-derived oxidant hypochlorous acid (HOCI), PMA, IL-8 or TNF-alpha (104, 105).

Some pathogens can stimulate the formation of ETs from macrophages, for example, macrophages grown in the presence of *M. tuberculosis* plus IFN-γ can induce ET formation (106). Cord-forming *M. tuberculosis*, which grows into organized structures on which bacteria remain attached in cord form, can also induce ET formation (107). The induction of ETs in macrophages by cord-forming *M. tuberculosis* was dependent on the virulence factor ESAT-6 (107). In addition, Aulik *et al.*, demonstrated, *in vitro*, that bovine alveolar macrophages and human macrophage cell lines TPH-1 and RAW 264.7 form macrophage ETs in response to hemolysins of *E. coli* and the leukotoxin of *Mannheimia haemolytica* (108).

*C. albicans* can simultaneously stimulate ET release and phagocytosis in macrophages (109). In this study, it was observed that the release of ET macrophages occurs before cell death (109). *In vitro*, placental macrophages generate ETs in response to *Streptococcus agalactiae*. These ETs contain metalloproteases, which may contribute to a weakening of the fetal membrane during infection (110).

The mechanisms by which ETs are induced in macrophages are not clear. For example, in placental macrophages, ET formation appears to be dependent on the production of ROS and actin polymerization (106). However, another study showed that inhibition of NADPH oxidase does not prevent ET formation (107). It has been suggested that the formation of ETs by NADPH oxidase independent mechanisms places intracellular calcium influx in a higher position (110).

ETs of macrophages have been associated with pathological processes such as autoimmunity and atherosclerosis. For example, ETs of synovial fluid macrophages are a resource of citrullinated histones that leading to formation of anti-citrullinated protein/peptide antibody in a murine model of autoimmune arthritis (111). In response to danger, ETs of different immune cells, including macrophages, have been reported to contribute negatively to making the plaque greater in patients with atherosclerosis (94).

#### Lymphocyte DNA Webs

T and B lymphocytes release DNA in response to PMA, ionomycin and serum from patients with Lupus or anti-immunoglobulin M plus LPS (45). In T-lymphocytes, activation with anti-CD3 and anti-CD28 antibodies triggers the release of DNA webs in a manner dependent on the production of ROS in the mitochondria (112). Another study reported that B-, T- and natural killer lymphocytes produce DNA webs made from mtDNA when stimulated with CpG oligonucleotide of class C. This mechanism was independent of the generation of ROS, cell death and signaling through the Toll 9 receptor. The DNA webs were devoid of microbicidal proteins, and when *E. coli* was cultured in the presence of B-cell DNA webs, no decrease in the number of colony-forming units was observed. In addition, B-cell DNA webs induce the release of type I interferon in peripheral blood mononuclear cells (113). Key features of ETs described in cells of the innate immune system, such as the presence of antimicrobial peptides, citrullinated histones, or the ability to trap bacteria or particles, have not been described in lymphocyte DNA webs. Further studies are needed to determine their similarity to ETs in other cells.

## Multiple Origins of Extracellular Traps

In the preceding sections we have reviewed the structural and functional similarities of ETs in different organisms, which raises the question as to whether this mechanism emerged early in the evolutionary history of life and all these ETs had a common evolutionary origin, that is, whether they are homologous. Inferences about homology have been proposed as a two-step process: first we look for a primary homology with the similarity test and then we test the homology hypothesis with the congruency test (114).

The similarity test states that structures should be morphologically, ontogenetically, or functionally similar, as well as exhibiting connectivity and structural correspondence (115, 116). There is no standard method for similarity testing, therefore, it is based on direct observation, conjecture and common sense (114, 117). The consistency test indicates whether the presumed homologous structures are consistent with other characteristics showing phylogenetic relationships between taxa (115).

We reviewed the evidence of ET formation in different groups to assess whether they are homologous characters. First, we proposed the primary homology from the review of the characteristics described ascribed to ETs. As detailed in the previous section, structural and functional similarities exist between the ETs of organisms such as protozoa, plants, invertebrates, and vertebrates, which has been noted previously by other authors (21, 118). Therefore, we propose that there is primary homology between ETs.

**Figure 1:**
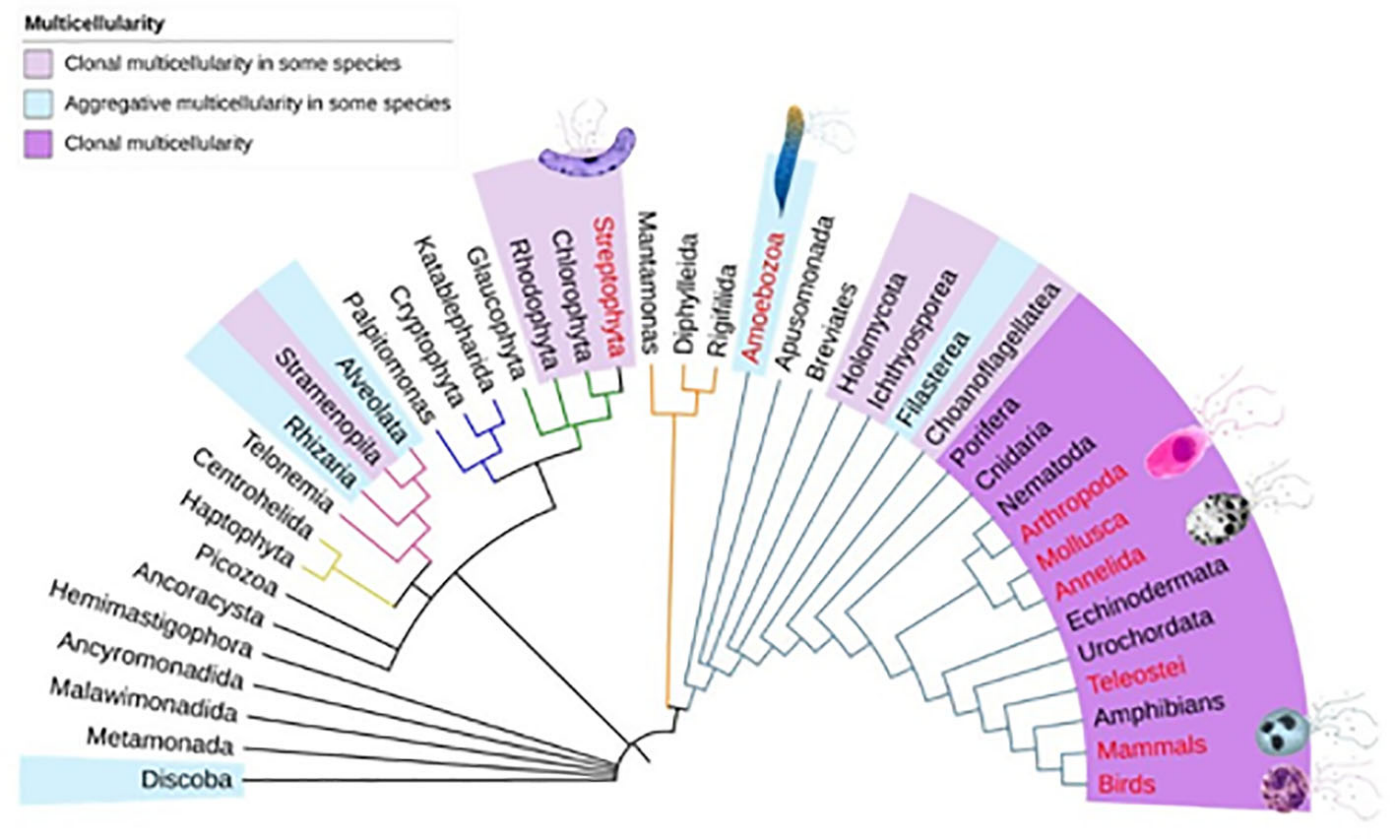
Phylogenetic relationships of species that form extracellular traps. The phylogenetic tree shows the major groups of eukaryotes (119–121). The positions of multicellular organisms and the positions of organisms that form extracellular traps (red) are shown.

Second, we used the congruency test to assess whether ETs could be considered a secondary homology. To perform the congruency test, we mapped the groups in which ETs have been described in the eukaryotic consensus phylogenetic tree (119–121). The phylogenetic tree shows that the presence of ETs is not a shared characteristic and that there is no congruence with the phylogenetic history of the groups, since the presence of ETs is observed in separate taxa (**Figure 1**). This leads us to reject the hypothesis of secondary homology. After phylogenetic analysis and a review of congruence, any pattern of non-homology is considered a result of homoplasy (when a trait has been gained or lost independently in separate lineages over the course of evolution) (122), Therefore, we contend that the formation of ETs is the result of homoplasy, and that ETs have multiple origins in the evolutionary history of living beings.

We determine that the formation of ETs may have originated several times in the evolution of eukaryotes. However, we cannot determine how many times they have originated due to two reasons. Firstly, we do not know all of the organisms that release ETs nor which ones do not release them within each group. Secondly, we cannot be sure whether the conditions for releasing ETs are ancestral or lost in some groups. However, it is unlikely that ETs are found in groups of eukaryotic unicellular organisms such as some species of unicellular fungi, choanoflagellates or chlorophytes. We suggest that ET formation has had at least three independent origins in embryophytes of the *Plantae* kingdom, in *amoebozoa* of the *Protozoan* kingdom, and in bilaterians. In the following paragraph we will discuss some of the reasons why the development of ETs is likely to be linked to multicellularity.

## Multicellularity and Extracellular Traps

The ability to release ETs has been closely linked to multicellularity, since the strategy of releasing DNA in defense against microorganisms is beneficial for a multicellular organism, although not for a unicellular one, from an evolutionary point of view (123). If a group of unicellular organisms were to develop cooperative immune responses such as the formation of ETs, unrelated organisms that do not contribute to defense would benefit from mutual cooperation. This is known as the “prisoner’s dilemma” (124). This population of opportunistic organisms would have an advantage over the cooperating organisms and their population would increase to a critical point at which, the entire population would eventually collapse (125). Therefore, the development of ETs must have originated in the first multicellular organisms.

Multicellularity has multiple origins in the evolution of eukaryotes, however, in order to determine the number of independent origins we must distinguish between clonal multicellularity and aggregative multicellularity. In clonal multicellularity the whole organism originates from a single cell, which divides mitotically, as in *Metazoa* and Embryophytes. In aggregative multicellularity, a set of unicellular organism groups form a multicellular organism at some stage of their development, as in *D. discoideum* (126). Regarding aggregative multicellularity, some authors suggest that the origins, have occurred between 16 and 22 times (127, 128). We have taken into consideration the groups that present a clonal multicellularity with more complex characteristics, such as intercellular communication, cell differentiation and tissue organization (in some cases), this had six origins in the history of eukaryotes (129).

Heterotrophic eukaryotes separated from the lineage that gave rise to Embryophytes and Chlorophylls more than 800 million years ago (129). Multicellularity in these groups appeared much later, ~650 million years ago for the metazoans and ~450 million years ago for the embryophytes (126). The ability to form ETs must have originated independently, after the groups were separated and multicellularity was established.

Previously, it has been suggested that there was a relationship between multicellularity and the presence of genes encoding for NADPH oxidases because these enzymes had only been identified in multicellular organisms (130). In addition, it was suggested that the time of origin of NADPH oxidases and multicellularity correlated with the time of origin of ETs, and that the presence of genes for NADPH oxidases would serve as a genetic signature in identifying which organisms may form ETs (123). However, there are reports showing that almost all groups of eukaryotic organisms, including single-cell protists, express NADPH oxidases (131).

The fungus *Saccharomyces cerevisiae*, which was thought not to express NADPH oxidases, expresses the yeast NADPH oxidase 1 (132). NADPH oxidases have even been reported in some prokaryotes, however, these NADPH oxidases are separate from eukaryotic NADPH oxidases in the phylogenetic tree, and have been suggested to be a subgroup of the NADPH oxidase family (133). Therefore, the hypothesis supporting a correlation between the presence of NADPH oxidases and ET formation should be discarded, although the hypothesis of a correlation between ETs and multicellularity still remains.

The development of multicellularity allows the differentiation of cells responsible for the defense of the organism, such as border cells, S cells and neutrophils. In unicellular organisms, defense mechanisms have been described as the secretion of antimicrobial peptides, the production of antibiotics and the interference of RNAs and restriction enzymes (134, 135). However, the transition to multicellularity requires the emergence of a more complex system of surveillance and protection that would allow recognition between self and non-self cells, as well as a tolerance of possible symbionts (136).

Now, we will make a brief review of the defense mechanisms in the cells of the immune system of the metazoans to illustrates the development of ETs is present in specialized cells for the defense of the organism once multicellularity is established.

Among the first processes related to the defense of the organism is phagocytosis, which appears in unicellular organisms as a feeding mechanism (137). It has been suggested that the origin of phagocytosis, similar to multicellularity, had independent origins in several eukaryotic lineages (138). The first cells specialized in the defense of the organism within the metazoan lineage, appear in the poriferous, and are known as amebocytes (139). These are responsible for phagocyting food particles and cellular debris (136). Other groups of invertebrates such as nematodes, annelids, mollusks, crustaceans and echinoderms have cells known as non-grained hemocytes, which phagocytize cells and foreign particles. These cells begin to present other innate immune defense mechanisms such as the secretion of antimicrobial peptides and the production of ROS, and some secrete ETs (140).

In arthropods, protochordates and vertebrates, hemocytes are present in granules containing enzymes and antimicrobial peptides (139–142). In many species of these organisms, it is already possible to distinguish between neutrophilic, eosinophilic and basophilic granulocytes (140). In granular hemocytes, the same mechanisms of the innate immune response, described in mammals as phagocytosis, antimicrobial peptide secretion, degranulation, ROS production, cytotoxicity and ET formation, can be observed (139, 143, 144). Therefore, the development of ETs occurs in specialized cells for the defense of the organism.

Fungi mainly present chemical defense responses such as toxin secretions, secondary metabolisms, peptides, antimicrobial proteins and ROS release (145–147). Although it has been shown that the secretion of these chemical mediators may be inducible through the detection of mechanical damage, bacterial peptidoglycan or pheromones (148–151), whether there is specificity in the detection of these signals, has not been shown. In the complete known sequences of fungi, homologous genes of the Toll-like receptors have not been encountered (146), although NOD-like receptors (NLR) are expressed. These last receptors perform hetero-incompatibility functions between different strains (152), although whether NLR receptors perform pathogen detection or damage signaling functions has yet to be demonstrated (146, 153). Even though there is no evidence of ETs in fungi, they are likely to be found since fungi are multicellular organisms.

## Conclusion

A defense against pathogens is essential for the survival of all organisms for which evolution has developed some remarkable mechanisms to combat different pathogenic microorganisms. Perhaps the most noteworthy of these defense mechanisms is the formation of ETs, in which bacteria, fungi and parasites are immobilized. The importance of this function is shown in the organisms of different, closely or distantly related taxonomic groups (protozoa, plants, arthropods, birds, marine and terrestrial mammals) in response to pathogens.

By means of the congruency test we have proved that the ETs in these organisms are examples of homoplasy and have multiple origins. Not all groups of organisms form ETs, and, of those that do not, the exact number of independent origins cannot be established. Furthermore, from the earliest stages in the evolution of ETs, the transition of organisms from the unicellular to the multicellular has been extremely significant.

## Author Contributions

Conceptualization: ERM, EPC, and MVJ. Writing—original draft preparation: ERM, LHG, IRM, EPC, and MVJ. Manuscript revision: ERM, LHG, IRM, LPCM, GILC, EPC, GMa, MTHH, and MVJ. All authors contributed to the article and approved the submitted version.

## Funding

This work was supported by National Technology of Mexico/IT Oaxaca (project number 8703.20-P) and Benito Juárez Autonomous University of Oaxaca. MVJ was funded by Dirección General de Asuntos del Personal Académico (DGAPA National Autonomous University of Mexico), UNAM (PAPIIT-IN201019).

## Conflict of Interest

The authors declare that the research was conducted in the absence of any commercial or financial relationships that could be construed as a potential conflict of interest.
